# The effects on clinical characteristics, potential factors and outcomes in SAPHO patients during the COVID-19 epidemic

**DOI:** 10.3389/fmed.2025.1580989

**Published:** 2025-07-16

**Authors:** Minhui Su, Haixu Jiang, Shengyan Liu, Pengfei Shen, Dinghua Zhou, Keqiang Zhu, Zixi Huang, Chen Li, Meiling Li

**Affiliations:** ^1^Department of Rheumatology, Changzhou Traditional Chinese Medical Hospital, Affiliated to Nanjing University of Traditional Chinese Medicine, Jiangsu, China; ^2^School of Chinese Materia, Beijing University of Chinese Medicine, Beijing, China; ^3^Peking Union Medical College Hospital, Chinese Academy of Medical Sciences & Peking Union Medical College, Beijing, China; ^4^Department of Orthopaedics, Changzhou Traditional Chinese Medical Hospital, Affiliated to Nanjing University of Traditional Chinese Medicine, Jiangsu, China; ^5^School of Life Sciences, Beijing University of Chinese Medicine, Beijing, China; ^6^Department of Rheumatology, Fangshan Hospital, Beijing University of Chinese Medicine, Beijing, China; ^7^Department of Hematology, The Second Affiliated Hospital of Fujian Medical University, Quanzhou, Fujian, China

**Keywords:** SAPHO syndrome, COVID-19, clinical characteristics, potential factors, crosssectional study

## Abstract

**Objectives:**

The coronavirus disease (COVID-19) pandemic has potentially impacted the care of patients with rheumatic diseases, including Synovitis, Acne, Pustulosis, Hyperostosis, and Osteitis (SAPHO) syndrome. We investigates the effects on clinical characteristics, potential factors, and outcomes in SAPHO patients during the COVID-19 pandemic.

**Methods:**

SAPHO patients were recruited for this cross-sectional study from Fangshan Hospital of Beijing University of Chinese Medicine. In total, 375 patients (mean age, 47.5 years, 72.53% females) were asked about demographic data, disease status, current treatments, and clinical manifestations during the epidemic, and potential relationships were analyzed.

**Results:**

Among 375 included patients, 329 were infected with coronavirus 2019. Compared with non-infected patients, infected ones were more likely to have higher disease activity (*p* = 0.006). However, Janus kinase (JAK) inhibitor use was lower in detected COVID-19 cases than in non-infected cases in our cohort. Disease symptoms during COVID-19 were more commonly present in the non-JAK group than JAK groups, including rhinorrhea (*p* = 0.030), nasal congestion (*p* = 0.023), sore throat (*p* = 0.042), pneumonia (*p* = 0.044), headache (*p* = 0.023), and prevalence of palpitation (*p* = 0.015). In this study, 29 participants underwent tonsillectomy. Tonsillectomized patients showed a significantly higher prevalence of pneumonia than patients who did not undergo tonsillectomy (*p* = 0.009). The associated effect factors were displayed in the case of previous tonsillectomy using multivariate analysis and Firth’s penalized likelihood. JAK inhibitor use (*p* = 0.025) and pneumonia (*p* = 0.011) were more likely to develop in patients with a history of tonsillectomy.

**Conclusion:**

Disease activity was inversely correlated with JAK inhibitor use in SAPHO patients with COVID-19 during the pandemic. Thus, JAK antagonists have protective effects on SAPHO patients with infections and can significantly mitigate new clinical crown symptoms. However, there was a significant negative correlation between tonsillectomy and the prevalence of SAPHO with COVID-19, which demonstrates that tonsillectomy may be associated with an increased risk of COVID-19 adverse outcomes, especially in cases of taste disorders and pneumonia.

## 1 Introduction

The coronavirus disease (COVID-19) pandemic, caused by severe acute respiratory syndrome coronavirus 2 (SARS-CoV-2), is an unprecedented global health crisis (1). SARS-CoV-2 is transmitted mainly through short-range air aerosols, respiratory droplets, and direct or indirect contact with infectious respiratory droplets (2). Since December 2019, SARS-CoV-2 has spread worldwide through droplets or direct contact, resulting in severe morbidity and mortality (3–5).

The COVID-19 outbreak, as a public health emergency of international concern, has had a major impact on the health of the public and other diseases such as rheumatoid arthritis (6). Many people with rheumatic diseases believe that they have been affected by the fight against COVID-19 (7). Some experienced feelings of loneliness that could exacerbate anxiety, depression, and the relapse of rheumatic diseases. In contrast, some experienced relief from disease activity. To date, the impact of COVID-19 on patients with Synovitis, Acne, Pustulosis, Hyperostosis, and Osteitis (SAPHO) syndrome is not fully understood. There are also limited data on which therapies or factors may magnify the susceptibility to infection and predict poor outcomes (3). We were curious about the true circumstances of the SAPHO population.

SAPHO syndrome is a rare inflammatory disorder that affects the skin, bones, and joints, indicating potential associations with autoimmune conditions (8). The COVID-19 pandemic has also brought great challenges to chronic disease management, especially for SAPHO patients. Furthermore, novel viral variant waves and seasonal outbreaks are expected to emerge in the near future. Hence, it is crucial, with growing attention, to clarify the effects of COVID-19 on patients with SAPHO syndrome.

In this study, we aimed to understand the clinical manifestations and analyze the latent effects and outcomes, such as previous tonsillectomy and medication application, in Chinese SAPHO patients during the course of the COVID-19 epidemic. These results will pave the way for further clinical analyses of SAPHO syndrome in combination with COVID-19.

## 2 Materials and methods

### 2.1 Participants

SAPHO patients were recruited for this cross-sectional study from Fangshan Hospital of Beijing University of Chinese Medicine (Beijing, China). We recruited patients who underwent at least one follow-up visit for our SAPHO syndrome cohort study. Participation in the study was voluntary, and written informed consent was obtained from each patient. An online orientation was provided to each participant before the questionnaire. This study complied with the Declaration of Helsinki and was approved by the Ethics Committee of Fangshan Hospital of Beijing University of Chinese Medicine.

The inclusion criteria were as follows: (1) diagnosis of SAPHO syndrome according to the diagnostic criteria proposed by Kahn in 2003 (9); (2) age 12–75 years; and (3) disease course > 1 month.

The exclusion criteria were as follows: (1) not cooperating to participate in the questionnaire; (2) filling an invalid questionnaire in which the data information is incomplete; (3) a diagnosis of cognitive impairment reported by the patient or caregiver, and (4) inability to answer one or more questions on the questionnaire. We use questionnaires and obtain the contact details in this study. What’s more, we conduct long-term follow-up for SAPHO patients. So we obtain information that these patients received the Sinovac COVID-19 Vaccine under the guidance of Chinese policies.

**FIGURE 1:**
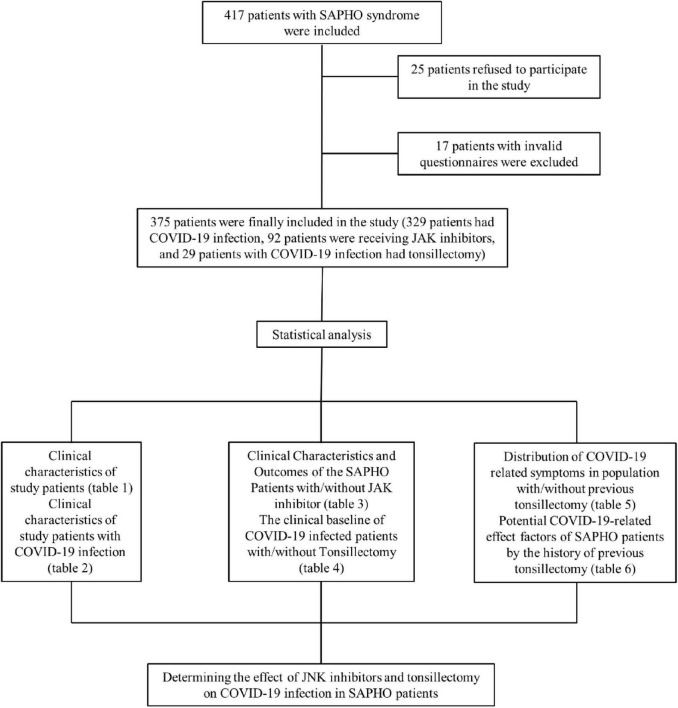
Flowchart of research methods and results.

A total of 417 patients were initially recruited and a total of 42 patients were excluded; therefore, a total of 375 patients participated in the study. Figure 1 shows the overall framework of this study.

### 2.2 Questionnaires

Questionnaires were distributed digitally on February 3, 2023, and collected on February 10, 2023. A total of 417 patients were sent questionnaires via telephone. Patients could complete the questionnaires independently by scanning a QR code. Demographic data, disease duration, current treatment, and clinical manifestations were also collected. Signs and symptoms were classified as COVID-19-related if they appeared *de novo* or as worsening of chronic symptoms within 14 days before or after a positive result of the microbiological test. Cronbach’s reliability coefficient was 0.939 (> 0.8 means the reliability of the test or scale is very good). KMO was 0.948(> 0.6 means the validity analysis of the test is very good).

### 2.3 Outcomes

In this study, the demographic data of patients with SAPHO syndrome and the clinical characteristics of COVID-19 in patients with SAPHO syndrome were clarified. However, the protective effect of Janus kinase (JAK) inhibitor therapy on SAPHO patients needs further clarification. Furthermore, the outcome was to determine the differences in the prevalence of the investigated signs and symptoms between patients who underwent tonsillectomy and those who did not. Factors affecting the development of signs or symptoms according to a history of tonsillectomy were assessed using multivariate analysis, together with patient characteristics.

### 2.4 Statistical analysis

All statistical analyses were performed using SPSS Version 26 (IBM Corp., Armonk, NY, United States). Continuous variables were expressed as medians and interquartile ranges, according to the data distribution determined using the Shapiro-Wilk test. Categorical variables were summarized as numbers and proportions (%). No imputation was performed for missing data. Fisher’s exact test was used to determine whether or not there is a significant association between two categorical variables. Continuous variables were compared using the Mann–Whitney U test. Logistic regression was performed in multivariate analysis. Thus, we employed logistic regression using Firth’s penalized likelihood for the issues of small sample size and parameter estimation bias, which provided a smaller confidence interval. Results of multivariate analysis are reported as odds ratios (ORs) and 95% confidence intervals (95% CIs). Statistical significance was set at *p* < 0.05.

## 3 Results

### 3.1 Study population

A total of 417 questionnaires were distributed in this study, of which 25 patients refused to participate, 17 had invalid questionnaires (incomplete data), and there were no deaths. Finally, 375 valid questionnaires were reviewed for completeness. The median age of the patients was 47.5 years, and 272 patients (72.53%) were females. SAPHO Patients had a disease duration of approximately 6 years. Among the 375 participants, 329 were infected with SARS-CoV-2, and the number of COVID-19 cases associated with pneumonia was 37. Thirty-five SAPHO patients previously underwent a tonsillectomy (Table 1).

**TABLE 1:** Clinical characteristics of study patients.

Variables		*N* = 375
Age(years), median (IQR)		47.50(18.00)
**Sex**		
	Male	103(27.47)
	Female	272(72.53)
BMI – median (IQR)		24.03(4.33)
**Smoking history, *n* (%)**		
	Current	80(21.33)
	Former	52(13.87)
	Never	243(64.80)
Current alcohol consumption, *n* (%)		84(22.40)
**Educational level, *n* (%)**		
	Illiterate/primary education	17(4.53)
	Lower/upper secondary education	151(40.26)
	Post-secondary education	207(55.20)
Presence of any allergy, *n* (%)		52(13.87)
**Any coexisting disorder**		
	None	247(65.87)
	Diabetes	26(6.93)
	Hypertension	59(15.73)
	Chronic cardiovascular disease	15(4.00)
	Chronic pneumopathy (e.g., asthma and COPD)	6(1.60)
	Chronic liver disease	15(4.00)
	Chronic renal disease	1(0.27)
	Cancer (current or previous)	8(2.13)
	Other diseases	46(12.27)
Disease duration of SAPHO (year), median (IQR)		6(6.00)
Active disease of SAPHO, *n* (%)		74(19.73)
**Baseline therapy, *n* (%)**		
	None	105(28.00)
	NSAIDs	139(37.07)
	DMARDs	30(8.00)
	CS	16(4.27)
	JAK inhibitor	94(25.07)
	Biological DMARDs	39(10.40)
Tonsillectomy, *n* (%)		35(9.30)
COVID-19 infection, *n* (%)		329(87.73)
Pneumonia, *n* (%)		37(9.87)

Data presented as the median (IQR) or number (%). Abbreviations: IQR, interquartile range; COPD, chronic obstructive pulmonary disease; n, number; NSAIDs, non-steroidal anti-inflammatory drugs; DMARDs, disease-modifying anti-rheumatic drugs; CS, corticosteroids; JAK inhibitor, Janus kinase inhibitor (tofacitinib); Biological DMARDs, biological disease-modifying anti-rheumatic drugs (TNFα inhibitors); Etanercept (28, 7.45%), Infliximab (4, 1.07%), Adalimumab (7, 1.87%); COVID-19, coronavirus disease 2019.

### 3.2 COVID-19 infected and non-infected population

**FIGURE 2:**
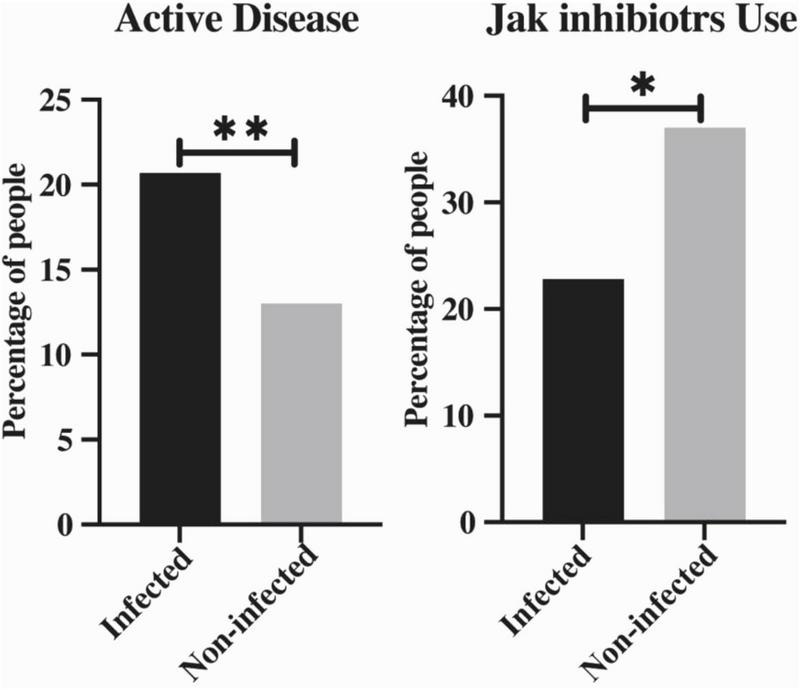
The percentage of active disease patients and JAK inhibitors users between infected and non-infected COVID-19 groups. Data were expressed as number (%). Fisher’s exact test was used to compare the outcomes between known groups. *p < 0.05, **p < 0.01, ***p < 0.001.

The median ages of COVID-19-infected and non-infected patients were 48 and 50 years, respectively (*p* = 0.813). Among all the SAPHO patients with viral infections, the proportion of females was higher than males (74.77% vs. 56.52%, *p* = 0.013) (Table 2). SAPHO is more common in women than in men (10, 11), and is predominant in middle-aged women (12, 13). To an extent, this is consistent with previous reports. We determined the frequency of patients infected and non-infected with COVID-19 based on JAK inhibitor drugs and disease activity. Compared to non-infected patients, infected patients were more likely to have higher disease activity (20.7% vs. 13.0%, *p* = 0.006) and lower use of JAK inhibitor drugs (22.8% vs. 37.0%, *p* = 0.044) (Figure 2).

**TABLE 2:** Clinical characteristics of study patients with COVID-19 infection.

Variables	COVID-19 infection		*p*
	Yes *n* = 329	No *n* = 46	
Age (years), median (IQR)	48 (18.00)	50(17.00)	0.813
Sex, *n* (%)			0.013*
Male sex	83 (25.23)	20 (43.48)	
Female sex	246 (74.77)	26 (56.52)	
BMI – median (IQR)	24.03(4.61)	23.70(2.99)	0.755
Smoking history, *n* (%)			0.822
Current	69 (20.97)	11 (23.91)	
Former	47 (14.29)	5 (10.87)	
Never	213 (64.74)	30 (65.22)	
Current alcohol consumption, *n* (%)	73 (22.19)	11 (23.91)	0.668
Presence of any allergy, *n* (%)	46 (13.98)	6 (13.04)	1.000
**Any coexisting disorder, *n* (%)**			
None	214 (65.05)	33 (71.74)	0.410
Diabetes	25 (7.60)	1 (2.17)	0.228
Hypertension	51 (15.50)	8 (17.39)	0.672
Chronic cardiovascular disease	14 (4.26)	1 (2.17)	1.000
Chronic pneumopathy (e.g., asthma and COPD)	6 (1.82)	0 (0.00)	1.000
Chronic liver disease	13 (3.95)	2 (4.35)	0.705
Chronic renal disease	1 (0.30)	0 (0.00)	1.000
Cancer (current or previous)	8 (2.43)	0 (0.00)	0.603
Other diseases	41 (12.46)	5 (10.87)	1.000
Disease duration (year), median (IQR)	6(6.00)	5(6.50)	0.554

Data presented as the median (IQR) or number (%). Fisher’s exact test was performed for Sex, Smoking history, Current alcohol consumption, Presence of any allergy, and Any coexisting disorder. Mann-Whitney U test was performed for Age, BMI, and Disease duration. n, number; IQR, interquartile range; BMI, Body mass index; COPD, chronic obstructive pulmonary disease. *p < 0.05.

### 3.3 The protective effects of JAK inhibitor therapy among SAPHO patients

Generally, SAPHO patients treated with JAK inhibitor drugs showed alleviated clinical symptoms. The participants were divided into two groups based on the use of JAK inhibitors. Of the included patients, 92 were administered JAK inhibitors. Respiratory symptoms during SARS-CoV-2 infection were more commonly presented in the non-JAK group than in JAK groups, included rhinorrhea (52.65% vs. 39.13%, *p* = 0.030), nasal congestion (57.24% vs. 43.48%, *p* = 0.023), sore throat (54.77% vs. 42.39%, *p* = 0.042), and pneumonia (4.35% vs. 11.66%, *p* = 0.044). A similar condition could be found in headache analysis between the two groups (56.18% vs. 42.39%, *p* = 0.023). No significant differences were observed in other respiratory symptoms between the two populations, such as cough (66.30% vs. 73.14%) and shortness of breath (32.61% vs. 44.17%), although there was a lower trend in JAK inhibitors use. JAK inhibitor users significantly presented with a lower prevalence of palpitation than those who did not use any JAK blocker drugs (30.43% vs. 45.23%, *p* = 0.015) (Table 3).

**TABLE 3:** Clinical characteristics and outcomes of the SAPHO patients with/without JAK inhibitor.

	JAK inhibitor		
	Yes *n* = 92	No *n* = 283	*p*
Fever, *n* (%)	62 (67.39)	216 (76.33)	0.100
Chills, *n* (%)	52 (56.52)	178 (62.90)	0.324
Cough, *n* (%)	61 (66.30)	207 (73.14)	0.232
Shortness of breath, *n* (%)	30 (32.61)	125 (44.17)	0.052
Rhinorrhea, *n* (%)	36 (39.13)	149 (52.65)	0.030*
Nasal congestion, *n* (%)	40 (43.48)	162 (57.24)	0.023*
Sore throat, *n* (%)	39 (42.39)	155(54.77)	0.042*
Myalgia, *n* (%)	49 (53.26)	172 (60.78)	0.223
Dizziness, *n* (%)	38 (41.30)	138 (48.76)	0.231
Headache, *n* (%)	39 (42.39)	159 (56.18)	0.023*
Nausea, *n* (%)	25 (27.17)	102 (36.04)	0.129
Diarrhea, *n* (%)	18 (19.57)	85 (30.04)	0.060
Appetite disturbance, *n* (%)	48 (52.17)	172 (60.78)	0.180
Taste disturbance, *n* (%)	41 (44.57)	138 (48.76)	0.548
Smell disturbance, *n* (%)	38 (41.30)	131 (46.29)	0.469
Palpitation, n (%)	28 (30.43)	128 (45.23)	0.015*
Insomnia, *n* (%)	10 (10.87)	47 (16.61)	0.152
Coagulation abnormalities, *n* (%)	0 (0.00)	4 (1.41)	0.576
Pneumonia, *n* (%)	4 (4.35)	33(11.66)	0.044*

Data presented as the number (%). Fisher’s exact test was used to compare the outcomes between known groups. *p*-values for each variable are reported. **p < 0.01.

### 3.4 The clinical baseline of the tonsillectomy SAPHO population infected with COVID-19

A total of 329 patients, including 29 tonsillectomized participants, were recruited for our investigation, and 246 patients (74.77%) were females. The median ages of the tonsillectomized and placebo patients were 44 and 49 years, respectively (*p* = 0.016). The therapeutic medications used varied in the population infected with SARS-CoV-2 with a previous tonsillectomy, including non-steroidal anti-inflammatory drugs, disease-modifying anti-rheumatic drugs, glucocorticosteroids, and JAK inhibitor drugs. Among all, patients after tonsillectomy did not desire to receive more medication than placebo patients (51.72% vs. 25.67%, *p* = 0.005). Moreover, this group received fewer JAK inhibitors drugs (3.45% vs. 24.67%, *p* = 0.009) (Table 4).

**TABLE 4:** The clinical baseline of the tonsillectomy SAPHO population infected with COVID-19.

		Tonsillectomy		
Variables	All patients *n* = 329	Yes *n* = 29	No *n* = 300	*p*
Age (years), median (IQR)	48(18.00)	44(17.00)	49(19.00)	0.016*
Sex				0.375
Male sex	83 (25.23)	5 (17.24)	78 (26.00)	
Female sex	246 (74.77)	24 (82.76)	222 (74.00)	
BMI-median (IQR)	24.03(4.61)	24.49(5.69)	24.02(4.33)	0.899
Smoking history, *n* (%)				0.463
Current	69 (20.97)	8 (27.59)	61 (20.33)	
Former	47 (14.29)	5 (17.24)	42 (14.00)	
Never	213 (64.74)	16 (55.17)	197 (65.67)	
Current alcohol consumption, *n* (%)	73(22.19)	11 (37.93)	62(20.67)	0.096
Presence of any allergy, *n* (%)	46 (13.98)	4 (13.79)	42 (14.00)	1.000
Disease duration (year), median (IQR)	6(6.00)	6(4.75)	6(6.00)	0.396
Active disease, *n* (%)	68 (20.67)	6 (20.69)	62 (20.67)	0.242
**Baseline therapy, *n* (%)**				
None	92 (27.96)	15 (51.72)	77 (25.67)	0.005**
NSAIDs	126 (38.30)	8 (27.59)	118 (39.33)	0.237
DMARDs	27 (8.21)	2 (6.90)	25 (8.33)	1.000
CS	14 (4.26)	0(0.00)	14 (4.67)	0.622
JAK inhibitor	75 (22.80)	1 (3.45)	74 (24.67)	0.009**

Data presented as the median (IQR) or number (%). Fisher’s exact test was performed for Sex, Smoking history, Current alcohol consumption, Presence of any allergy, Active disease, and Baseline therapy. Mann-Whitney U test was performed for Age, BMI, and Disease duration. *p*-values for each variable are reported. *p < 0.05, **p < 0.01. n, number; IQR, interquartile range; NSAIDs, non-steroidal anti-inflammatory drugs; DMARDs, disease-modifying anti-rheumatic drugs; CS, corticosteroids.

### 3.5 Distribution of COVID-19-related symptoms in population with/without previous tonsillectomy

The distribution of the examined signs and symptoms according to previous tonsillectomy and related univariate analysis are summarized in Table 5. Tonsillectomized patients showed a significantly higher prevalence of pneumonia compared to patients who did not undergo tonsillectomy (27.59% vs. 9.67%, *p* = 0.009). Although tonsillectomized patients showed no distinct differences in taste disturbance, multivariate logistic regression analysis showed a *p-*value of 0.051 < 0.1. Tonsillectomized patients did not show an increased risk of developing other signs and symptoms, such as fever, chills, cough, shortness of breath, rhinorrhea, nasal congestion, sore throat, myalgia, dizziness, headache, nausea, diarrhea, appetite disturbance, palpitation, insomnia, and coagulation abnormalities (Table 5).

**TABLE 5:** Distribution of COVID-19-related signs and symptoms.

Tonsillectomy				
Signs and symptoms	All patients *n* = 329	Yes *n* = 29	No *n* = 300	*p*
Fever, *n* (%)	51 (15.50)	25 (86.21)	26 (86.67)	1.000
T > 39°C, *n* (%)	46 (13.98)	3 (10.34)	43 (14.33)	0.780
Chills, *n* (%)	230 (69.90)	21 (72.41)	209 (69.67)	0.835
Cough, *n* (%)	268 (81.46)	23 (79.31)	245 (81.67)	0.802
Shortness of breath, *n* (%)	155 (47.11)	12 (41.38)	143 (47.67)	0.563
Rhinorrhea, *n* (%)	185 (56.23)	18 (62.07)	167 (55.67)	0.561
Nasal congestion, *n* (%)	202 (61.40)	16 (55.17)	186 (62.00)	0.550
Sore throat, *n* (%)	194 (58.97)	14 (48.28)	180 (60.00)	0.240
Myalgia, *n* (%)	221 (67.17)	22 (75.86)	199 (66.33)	0.408
Dizziness, *n* (%)	176 (53.50)	19 (65.52)	157 (52.33)	0.242
Headache, *n* (%)	198 (60.18)	19 (65.52)	179 (59.67)	0.692
Taste disturbance, *n* (%)	179 (54.41)	21 (72.41)	158 (52.67)	0.051
Smell disturbance, *n* (%)	169 (51.37)	18 (62.07)	151 (50.33)	0.249
Palpitation, *n* (%)	156 (47.42)	14 (48.28)	142 (47.33)	1.000
Pneumonia, *n* (%)	37(11.25)	8(27.59)	29(9.67)	0.009**

Data presented as the number (%). Fisher’s exact test was used to compare the outcomes between known groups. *p*-values for each variable are reported. **p < 0.01.

### 3.6 Potential COVID-19-related effect factors of SAPHO patients by history of tonsillectomy

Table 6 summarizes the results of the multivariate analysis, showing the associated effect factors at potential risk of developing in cases of previous tonsillectomy. The use of JAK inhibitors (*p* < 0.05), taste disturbance (*p* < 0.05), and pneumonia (*p* < 0.05) more likely occurred in the patients who had a history of tonsillectomy (Table 6). Thus, Table 7 shows the results of logistic regression by using Firth’s penalized likelihood for parameter estimation. The results in Table 7 are similar to those in Table 6 except for taste disturbance.

**TABLE 6:** Potential COVID-19-related influencing factors affected by history of previous tonsillectomy.

Characteristic	Tonsillectomy	*p*
Age (years)	0.95 (0.91–0.98)	0.004**
JAK inhibitor (No vs. Yes)	10.29 (1.33–79.52)	0.025*
Taste disturbance (No vs. Yes)	0.40 (0.16–0.98)	0.045*
Pneumonia (No vs. Yes)	0.30 (0.11–0.78)	0.013*

Results of the multivariate logistic regression. Odds ratio (95% confidence interval) and *p*-values for each variable are reported. *p < 0.05, **p < 0.01.

**TABLE 7:** Potential COVID-19-related influencing factors affected by history of previous tonsillectomy.

Characteristic	Tonsillectomy	*P*
Age (years)	0.95 (0.92–0.98)	0.003**
JAK inhibitor (No vs. Yes)	6.86 (1.28–36.80)	0.025*
Taste disturbance (No vs. Yes)	0.42 (0.18–1.00)	0.050
Pneumonia (No vs. Yes)	0.30 (0.12–0.76)	0.011*

Results of the multivariate logistic regression using Firth’s penalized likelihood. Odds ratio (95% confidence interval) and *p*-values for each variable are reported. *p < 0.05, **p < 0.01.

## 4 Discussion

The coronavirus disease (COVID-19) pandemic has spawned a health crisis that inevitably affects patients with SAPHO syndrome. Little data are available in the literature on SAPHO patients with COVID-19. In our survey, we presented a detailed analysis to explore the influence of COVID-19 on SAPHO patients, especially for reporting reactions after medication treatment.

The study population was divided into infected and non-infected groups, indicating that the incidence of uncontrolled disease activity was much higher in infected patients during the COVID-19 pandemic. This outcome is in accordance with previous studies showing that patients with pre-existing rheumatic diseases may flare or develop novel autoimmune features during SARS-CoV-2 infection (14, 15). Although the precise mechanism of COVID-19 triggering SAPHO syndrome is still a mystery, rising interleukin and TNF-α levels, which can be caused by the virus, are mentioned in the pathogenesis of SAPHO syndrome (16, 17). Moreover, JAK inhibitors have the ability to suppress those interleukins (such as IL-6) and TNF-α, which may explain the low disease activity level observed in JAK blocker recipients in our research. This finding is helpful in showing the relevance of the two sides, which still needs to be investigated in ongoing clinical trials.

Despite the theories mentioned above regarding the effect of JAK inhibitors, there are concerns about whether the use of background immunosuppressive medications, such as JAK inhibitors, will temper the risk of clinical manifestations and severe outcomes in individuals with SAPHO who have COVID-19. Significant clinical experience has been accumulated regarding the use of JAK inhibitors for treating COVID-19 and the associated inflammatory status (17, 18). In this study, we also present proof to remarkably strengthen this result. Hence, it appears that JAK inhibitors exert protective effects in SAPHO patients with COVID-19.

There is currently some evidence that tonsillitis, a possible predisposition to SAPHO syndrome, may be associated with improved bone and skin symptoms in SAPHO patients (15). Additionally, SAPHO syndrome with tonsillectomy, in terms of COVID-19-related adverse outcomes, was revealed by further probing the characteristics of previously tonsillectomized patients. Younger participants seemed to accept this procedure. Studies have been conducted to determine whether tonsillectomy aggravates the severity of COVID-19; however, the results are not consistent. Some studies have suggested that tonsillectomy increases the incidence of fever, chills, and malaise in patients with COVID-19. The clinical management of patients requires greater caution.

Moreover, data regarding specific risk factors in tonsillectomized SAPHO patients are yet to be determined. As mentioned above, a history of tonsillectomy may negatively affect the risk of pneumonia and taste disturbance. Pneumonia partly reflects more intense systemic involvement. Tonsillectomized patients still show a greater risk of expressing a more intense systemic response to SARS-CoV-2, lacking the protective effect of tonsils through the distribution of dendritic cells, B-regulatory cells and effector T-cells (19). In addition, bilateral tonsillectomy may be beneficial for the treatment of arthralgia in patients with SAPHO syndrome (20), which may be a harmful factor for taste dysfunction. We have not previously noticed this situation. Interestingly, we initially used regression analysis to obtain a positive result. But we supplemented the logistic regression analysis using Firth’s penalized likelihood for the issues of small sample size and parameter estimation bias, which showed that taste disorders were negative results. In view of this contradiction, we consulted some literature. According to previous reports (21–23), the incidence of dysgeusia after surgery is estimated to be 10–20% (24). This occurrence was more frequent than expected. Women and patients aged < 60 years had a significantly higher rate (25). There are several reasons for taste disturbance after tonsil surgery. Zn levels were found to be a probable reason. Another probable cause is a surgical injury to the lingual branch of the glossopharyngeal nerve. However, the frequency, time course, and prognosis of long-lasting dysgeusia after tonsillectomy remain unclear. Therefore, we could not determine whether the rising taste dysfunction rate was a long-term problem after tonsillectomy or a symptom of COVID-19.

Pneumonia and taste dysfunction require more attention when determining the management of SAPHO syndrome during the COVID-19 pandemic, which may not end in a short period. Further studies with larger sample sizes focusing on these two symptoms are needed to verify the above risks in treating patients with SAPHO syndrome.

The main limitation of this study is the absence of deceased patients in our cohort, which may have affected the analysis of disease severity according to the variables investigated. This was unavoidable because of the impossibility of retrieving complete information regarding previous surgeries, coexisting disorders, and COVID-19-related symptoms. However, this was deemed acceptable at the time of the study design given the small percentage of deaths in relation to the total number of cases in China during the recruitment phase, probably with low effects on the outcomes.

Another limitation is that we included a limited number of patients, particularly those who underwent tonsillectomy. Therefore, we were unable to conduct more sophisticated analyses to control for potential confounding effects. We were unable to investigate the blood and imaging outcomes of the infected patients. Nevertheless, pneumonia is characterized by the discovery of imaging abnormalities related to a new crown in the lungs. Therefore, we lacked a classification of pneumonia on computed tomography (CT). Although we observed a difference in the incidence of pneumonia between the tonsillectomy and non-tonsillectomy groups, there may be cases in which patients who did not undergo tonsillectomy were unwilling to undergo Chest CT because of the lack of apparent symptoms. A more inclusive cohort accounting for all the above mentioned biases, factors, and possible indicators of severity may be necessary to clarify the possible influence of SARS-CoV-2 and its variants on patients with SAPHO in China.

This study has some limitations. On the one hand, participants may be unwilling or afraid to provide honest answers for some reason or provide false information out of certain motives, resulting in biased answers; on the other hand, participants may be affected by certain factors (such as personal emotions or social environment), which leads to response bias in the data. Poorly designed questions may lead to inaccurate or incomplete responses from participants or introduce bias or misleading results in question formulation. In addition, the language and cultural backgrounds of the participants could affect the accuracy of the data. In response to the above problems, we took measures to make the research results as accurate and scientific as possible. Regarding possible answer and reaction bias in the questionnaire results, we actively provided psychological counseling to the participants and protected their personal privacy. Simultaneously, we consulted a large number of studies and adopted many studies on other diseases, making the questionnaire design more scientific. Additionally, for participants with low cultural backgrounds or language barriers, we carefully interpreted the questionnaire questions and invited family members to participate in the survey. The above measures made our investigation and research more scientific and standardized.

## 5 Conclusion

To the best of our knowledge, the main strength of this study is that it is the first to investigate the clinical characteristics, potential factors, and outcomes of SAPHO patients infected with SARS-CoV-2 in mainland China. According to our results, disease activity was inversely correlated with the use of JAK inhibitors in this special population during the epidemic. Thus, JAK antagonists have protective effects on SAPHO patients with viral infections and can significantly alleviate new crown symptoms. However, there was a significant negative correlation between tonsillectomy and the prevalence of SAPHO in patients with COVID-19, which demonstrates that tonsillectomy may be associated with an increased risk of COVID-19 adverse outcomes, especially in cases of taste disorders and pneumonia. Therefore, the use of JAK inhibitors and tonsillectomies in SAPHO patients should be considered for their effects on COVID-19. This study provided new insights into the treatment of patients with SAPHO during the COVID-19 pandemic.

## Data Availability

The original contributions presented in the study are included in the article/supplementary material, further inquiries can be directed to the corresponding author/s.
